# Threats to Mental Health Facilitated by Dating Applications Use Among Men Having Sex With Men

**DOI:** 10.3389/fpsyt.2020.584548

**Published:** 2020-11-13

**Authors:** Katarzyna Obarska, Karol Szymczak, Karol Lewczuk, Mateusz Gola

**Affiliations:** ^1^Institute of Psychology, Polish Academy of Sciences, Warsaw, Poland; ^2^Institute of Psychology, The Maria Grzegorzewska University, Warsaw, Poland; ^3^Institute of Psychology, Cardinal Stefan Wyszyński University, Warsaw, Poland; ^4^Swartz Center for Computational Neurosciences, Institute for Neural Computation, University of California, San Diego, San Diego, CA, United States

**Keywords:** MSM (men who have sex with men), dating applications, mental health, compulsive sexual behavior disorder (CSBD), chemsex, substance (ab)use, risky sexual behaviors

## Abstract

In the last years, dating applications (DAs) have had a significant impact on the way in which people seek sexual and romantic relationships. Social groups, such as men having sex with men (MSM), who can experience discrimination and social isolation, find DAs especially engaging and helpful in finding sexual partners. Previous studies have provided evidence showing vulnerability to mental health problems among the MSM population—these problems can be potentially facilitated by DAs use. Excessive use of DAs is associated with lower well-being and life satisfaction, depression, higher substance use, and lower sleep quality. Therefore, there is a need for a better understanding of psychological functioning and risk factors associated with the use of DAs among MSM, which we focus on in this review. We also discuss two relatively new research areas: compulsive sexual behavior disorder and chemsex, and their relation to geosocial-networking mobile technologies. Finally, we point out the limitations of available studies on the mental health of MSM using DAs and propose further research directions.

## Introduction

In recent years, mobile dating applications (DAs) have become popular worldwide, changing the way people establish intimate relations, and seek sexual partners. Although a comparable number of both women and men (1) use geosocial-networking mobile applications for dating, there is a category of “apps” dedicated specifically for non-heterosexual males (2) such as Grindr, Romeo, Hornet, or Adam4Adam.

In this narrative review, we present (in section Characteristics and Mental Health of MSM Who Use Mobile DAs) the current state of knowledge on sociodemographic and mental health of men having sex with men (MSM) using the mentioned applications, presenting both the advantages (lower stigmatization, increased partner availability) and threats (e.g., exposure to risky sexual behaviors) associated with DAs use. Then, we point to emerging and socially important issues such as (in section Substance Abuse and Sexualized Drug Use Among MSM Who Use DAs) sexualized drug use [SDU; (3)], also labeled as “chemsex,” and (in section What Do We Know About CSBD Among MSM Who Use DAs) compulsive sexual behavior disorder [CSBD; (4)], which have not been fully examined yet in association with MSM DAs users. Finally (in section Discussion), we discuss the limitations of available studies and propose directions for future research.

## Methods and Materials

### Literature Searching Description

For the purpose of this literature review, we have searched Google Scholar databases for scientific papers published in peer-reviewed journals. In total, we retrieved 4,270 articles published between 2010 and 2020 (the search was conducted in June 2020). The keywords used in the database search included “men having sex with men” and “mental health.” After the exclusion of studies regarding HIV infection, only 189 articles remained. Further, we narrowed the scope to DAs, which resulted in 59 articles, most of which we present in this narrative review. The titles and abstracts of the retrieved articles were evaluated, and the eligible articles were selected for full-text review. Particular manuscripts were included if (a) studies focused on MSM group, (b) studies focused on online dating and geosocial networking applications use, (c) studies focused on mental health issues and psychosocial consequences associated with DAs use, or (d) articles were published in English. Articles were excluded if (a) studies focused mainly on sexual health (promoting sexual health, HIV, and other STDs prevention) or (b) manuscript was based on a case study, observational study, or qualitative study.

#### Characteristics and Mental Health of MSM Who Use Mobile DAs

The difficulties in finding a romantic or sexual partner in a mainly heteronormative society are, to a large degree, alleviated in cyberspace, where LGBT communities can receive support and engage in relationships more easily (5). Online dating has become a remedy for low partner availability, social isolation, and discrimination (6).

Research has shown that homonormative people experience a lack of tolerance or acceptance, and as many as 20% of them are insulted due to their sexual orientation (7). This can contribute to higher levels of minority stress and stigmatization, which are in turn associated with a higher risk for a range of mental health disorders (8). Moreover, depression is linked to minority stressors in LGBT populations (9). Deficiency of social support, victimization, and exposure to violence have a significantly stronger correlation with poorer mental health in the LGBT group compared with the heterosexual group (10). Research (11) conducted on an LGBT and heterosexual representative sample (*n* = 222,548) showed that non-heterosexual participants, in comparison with heterosexual ones, experience a higher level of stress over a lifetime and their attachment to local society is weaker. Available research indicates that, relative to their heterosexual counterparts, homosexual and bisexual males are 1.5–3 times more vulnerable to depression, anxiety, and substance use disorders (12), as well as more likely to attempt suicide (13). Homonegativity contributes to consequences in the mental health of MSM, for example, in the form of adverse effects on well-being (14), low self-acceptance, and loneliness (15).

Due to the social marginalization of MSM groups, access to DAs provides a platform for establishing satisfying social and sexual relationships (16) and an outlet for sexual expression in which the threat of being a target of prejudice, stereotypes, and stigmatization is lowered (6). The high prevalence of DAs use, in conjunction with high rates of mental health disorders in the MSM group, may be why this group is the most often studied in terms of online dating.

To the best of our knowledge, there are two systematic reviews (17, 18) investigating sociodemographic characteristics and risky sexual behaviors among MSM using geosocial networking applications. MSM is a relatively small population [5–7% of males; (16)]. Both Anzani et al. (18) as well as Zou and Fan (17), indicate that the mean age of DAs users ranges between 25 and 35 years, and compared with non-users, they have a higher level of education and income and reported a greater number of sexual encounters in the last few months and in a lifetime perspective. Landovitz et al. (19) concluded that up to 56% of MSM DAs users met sexual partners in the previous 3 months only via Grindr (the most popular app). Non-heterosexual men also constitute the most active group using DAs to hook up for sexual purposes (18). MSM using DAs engage in unprotected anal intercourse (both receptive and insertive) with partners of unknown HIV status more frequently than non-app users, usually under the influence of drugs or alcohol during sexual activity (18).

The vast majority of studies (17, 19, 20) on MSM app users are more focused on sexual health, especially on HIV and prevalence and prevention of other STDs, than on mental health. Recent research (6) on Grindr users shows that excessive use of DAs is linked to lower psychological and social well-being, and some participants reported addictive symptoms over extended time use. Zervoulis (2) confirmed that heavy use of DAs is correlated with higher isolation, lower perception of community belonging, and less satisfaction of life. Duncan et al. (21) found that MSM app users reported low sleep quality (34.6% of respondents) and short sleep duration (43.6% of respondents), which were linked to depressive symptoms, engaging in unprotected anal sex, as well as alcohol and drug use. Moreover, loneliness seemed to be negatively correlated with sharing private information through gay DAs (2). In contrast, a positive impact on sexual self-acceptance could be observed in the LGBT group of people who were digitally connecting to each other (22). MSM who mainly seek sexual partners using DAs experience a higher level of confidence and satisfaction with life than men seeking non-sexual relationships. In a group of MSM who are looking for other than sexual relations (e.g., romantic relationship or friendship), using DAs may also lead to frustration due to an unrealized need for intimacy (2).

Sexual sensation seeking (SSS), defined as a drive for thrilling novel sexual experiences (23), has been shown to be a strong correlate of risky sexual behaviors (23–25). A high intensity of SSS is positively correlated with a higher number of sexual partners met via DAs, a higher likelihood of being HIV-positive, as well as a greater amount of anal intercourse, including intercourse without condoms and in the receptive position (23–25). The moderating role of SSS in a relationship between internet use and high-risk sexual behaviors in the MSM group has been identified (20). SSS has also been found to be a moderator between using alcohol or drugs before sexual activity and higher rates of unprotected anal intercourse among MSM (26).

#### Substance Abuse and Sexualized Drug Use Among MSM Who Use DAs

Another relatively well-studied aspect of MSM's mental health is substance abuse, especially during sexual activity. Recreational drug use in the MSM group is more common than in the general population (8), as taking psychoactive substances may be an experimental response to or a coping strategy for social marginalization (27). Non-heterosexual males are 1.5–3 times more vulnerable to alcohol dependence and illicit substances use compared with the heterosexual male population (12). Studies showed that 30% (28) or even 48% (19) of app-using MSM had been under the influence of alcohol and/or drugs during sex in the past month. App-using MSM in comparison to non-app using MSM, reported a 59.3–64.6% higher rate of cocaine, ecstasy, methamphetamine, and injection drug use, as well as a high rate of binge drinking in a lifetime (29, 30). The MSM community is more likely to engage in sexualized drug use (SDU). SDU is also known as “chemsex,” defined as any use of specific (e.g., methamphetamine, ecstasy, GHB) drugs before or during planned sexual activity to facilitate, initiate, prolong, sustain, and intensify the sexual encounter (31, 32). A recent review (32), based on 28 studies, estimates the prevalence of engaging in chemsex among MSM between 4 and 43% depending on the assessed population (ranging from clinical settings to urban areas).

Chemsex is associated with engaging in lengthy sex sessions and with a larger number of casual partners with an unknown HIV status (33). A combination of needle sharing, condomless sexual behaviors and being under the influence of drugs enhances the transmission of STDs (34). The fact that chemsex is associated with adverse mental health outcomes and may cause negative psychosocial consequences is an issue for concern (35). Some reports (31, 36, 37) described situations where MSM chemsex participants experienced severe psychological distress, psychotic symptoms, short-term depression, anxiety, long-term memory loss, and personality changes.

Studies show that it is quite common among MSM to use apps not only to engage in sexual activities, but also for sex parties, often associated with drug taking (38). For example, in Thailand, 73% of the MSM community use DAs for sexual purposes, as well as for inviting partners into illicit drug practice, with a 77% effectiveness of invitation rate (39). Latest review (40) provides data showing that MSM use geosocial network applications (a) to acquire drugs before engaging in sexualized drug use, (b) to sell sex in exchange for drugs, (c) to arrange sex with someone they would not have had sex with when sober, and (d) to find substance-using partners. Patten et al. (40) concluded that there is a mutual relationship between engaging in chemsex and using DAs among MSM.

**Figure 1:**
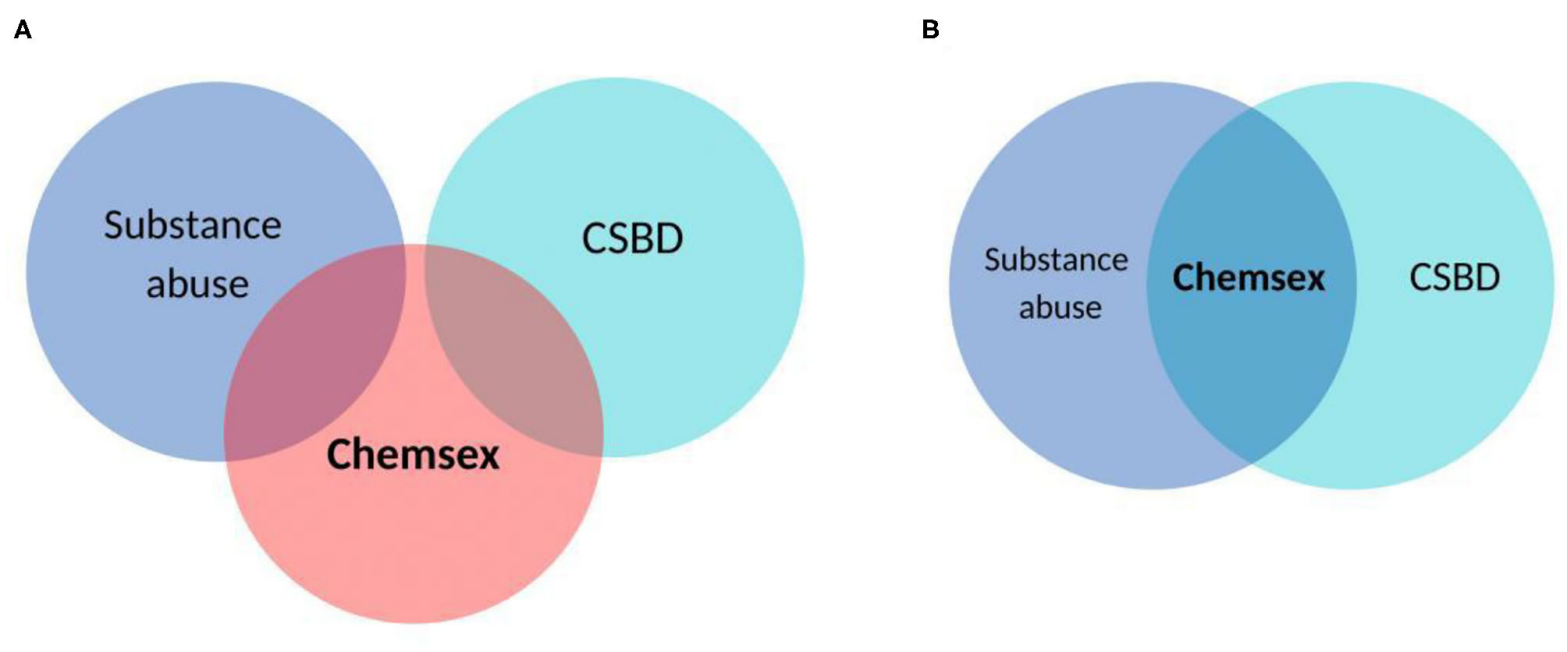
The presentation of chemsex as a separate entity **(A)** and as conjunction of substance use disorder and compulsive sexual behavior disorder **(B)**.

Although chemsex is a social concept, it may be considered a new form of addiction to sexual experiences induced and enhanced by psychoactive substances and facilitated by geosocial network applications. Future studies should examine if chemsex could be conceptualized as a conjunction of substance use disorder and compulsive sexual behavior disorder (see Figure 1) or a completely separate entity.

#### What Do We Know About CSBD Among MSM Who Use DAs

Compulsive sexual behavior disorder (CSBD), included recently in the 11th revision of the International Classification of Disorders (ICD-11) published by the World Health Organization (4), is characterized by a behavioral pattern in which a person (a) engages in repetitive sexual activity that has become a central focus of his/her life to the point of neglecting health and personal care or other interests, activities, and responsibilities; (b) has made numerous unsuccessful efforts to control or significantly reduce repetitive sexual behavior; (c) continues to engage in repeated sexual behavior despite adverse consequences; and (d) continues to engage in repeated sexual behavior even when he/she derives little or no satisfaction from it (4). The most common behavioral manifestation of CSBD is problematic pornography use accompanied by compulsive masturbation, and recent representative self-reported studies in the USA (41) and Poland (42) indicate that 9–11% of men and 3% of women, regardless of sexual orientation, perceived themselves as addicted to pornography. Compulsive use of paid sexual services or risky casual sexual encounters are also common among individuals meeting CSBD criteria (43).

Recognition of CSBD in ICD-11 raises a question regarding its prevalence among the MSM community and specifically among MSM using DAs. Unfortunately, CSBD has not been fully studied in the MSM community so far. Publications on the general population found a positive association between using geosocial networking applications and CSBD, showing that users of geosocial-network applications (compared to the general online population) are more likely to be young, non-heterosexual males. However, results of a recent study (44) on users of geosocial-networking applications contradict most earlier findings and suggest that the popularity of such applications increased among heterosexual populations.

Nonetheless, the majority of the data suggest DAs are more popular among MSM than among other groups, and their frequent use may potentially constitute a risk factor for CSBD development. Namely, it is possible that DAs may facilitate sexual encounters and novelty seeking in the sexual domain (especially among individuals with high sexual sensation seeking), potentially contributing to the development of CSBD at least in some subjects. A reverse relation is also possible: individuals with CSBD may be more likely to use DAs because they facilitate sexual encounters. This underdeveloped research area is of high importance, as among MSM who met sexual partners via the Internet, CSBD is associated with a higher frequency of engaging in HIV sexual risk behaviors (45).

The clear diagnostic criteria of CSBD described in ICD-11 (4) will facilitate future research on this behavioral pattern among MSM, which in turn will hopefully result in obtaining a detailed picture of interactions between CSBD, substance use disorders and such phenomena as chemsex and DAs use among the MSM community.

## Discussion

In this narrative review, we aimed to present findings on research examining mental health among MSM using DAs. We focused mainly on aspects associated with substance use and risky sexual behaviors as MSM seem to be especially vulnerable to threats in this domain. Available data on mental health primarily describe the prevalence of mental disorders (depression, anxiety, personality disorders) among MSM. In short, these data show that, compared with non-users, MSM using DAs report lower perception of community belonging, higher isolation, less satisfaction with life, and worse quality of sleep (2, 21). The stigma and discrimination experienced by the MSM community may be a possible explanation for the more frequent recreational drug use in this group than in the general population. Additionally, based on previous studies reviewed above, it seems that risky sexual behaviors among MSM using DAs are inseparable from substance abuse. DAs may facilitate seeking sexual partners, and off-line sexual encounters are frequently accompanied by drug use. Sexualized drug use may be associated with an increased risk of polydrug substance abuse, risky sexual behaviors, transmission of STDs, severe psychological distress, short-term depression, anxiety, and even psychotic episodes or changes in personality (35). Currently, little is known about the prevalence of CSBD among MSM DAs users, and it remains unclear to what extent chemsex is associated with CSBD and whether it can be understood as a behavioral pattern standing at the conjunction of CSBD and substance use disorders. Available data (44) suggest that frequent use of DAs could be a risk factor for CSBD. The sexual sensation seeking may be a crucial correlate and even lead to the development of both CSBD and sexualized drug use. On the other hand, for individuals with already developed CSBD, geosocial-network apps may provide an unlimited source of sexual partners and novel experiences.

Several gaps in knowledge should be noted with respect to current studies on psychological and sexual functioning of MSM using DAs, and they should be considered important goals for future investigations (see Table 1).

**Table 1:** Recommendations for future studies on mental and sexual health among DAs users.

**Research area**	**Goals**
Mental health	To explore the positive impact of DAs use on mental health and social functioning among MSM. Further examination of adverse mental health consequences associated with engaging in relationships through online DAs among MSM.
Chemsex	To investigate the relations between chemsex, CSBD and substance use disorders. To consider if chemsex could be treated as a new form of addiction to sexual experiences, engaged in under the influence of specific drugs. To examine the nature of sexualized drug use among women who have sex with women (WSW) and among the heterosexual population using DAs. To assess the prevalence of sexual dysfunction and sexual concerns beyond HIV and STDs infection among apps using MSM chemsex participants. To examine if experiencing CSBD symptoms may lead to using DAs more often, which can lead to further development of symptoms and more frequent engagement in Chemsex.
CSBD	To explain whether DAs are a way to develop symptoms of CSBD because of the availability of sexual stimuli and sexual partners. To assess the prevalence of CSBD among app users MSM and association between CSBD and SSS.

It is also important to mention that mobile applications can be used to promote mental health, as well as for prevention or therapeutic programs (46). Ameri et al. (47) indicated that short-term interventions based on mobile phone applications and texting could decrease the rate of methamphetamine use, condomless anal intercourse, and HIV transmission among MSM. Another example of a harm reduction intervention of sexualized drug use is the German app “C: KYL” (“Chems: Know Your Limit”). C: KYL aims to reduce the risk of severe negative consequences such as dissociation and overdose through monitoring of drug-taking during chemsex sessions. Overall, mHealth strategies have a positive influence on health-promoting behaviors, appointment attendance, and accessibility to information and may present an effective means for mental health promotion and prevention if they provide optimized strategies for the MSM group (48, 49).

## Limitations

This review is a preliminary investigation that highlights associations of DAs use and mental health issues among MSM. However, important limitations of the current work should be noted. First, there is a limited number of studies on the psychological functioning of MSM using DAs. This is especially true for CSBD, which is a new diagnostic unit. The vast majority of previous research examined the aspects of promoting sexual health, as so far, the primary need in the MSM group was prevention of HIV and other STIs. Second, our review encompasses studies focusing only on the group of non-heterosexual males. Mental health threats posed by DAs among heterosexual men as well as women fell outside the scope of the current manuscript. Third, the use of apps and social media for mental health promotion and prevention of mental disorders is not a focus of our analysis. Future studies should also examine the unique opportunities for mental health promotion that dating (and other) applications, as well as social media and social networking platforms, bring [see (50)]. Lastly, our hypothesis that chemsex may be a conjunction of CSBD and substance use has yet to be validated. This hypothetical assumption should be taken as an inspiration and invitation to future research.

## Conclusions

Primary mental health difficulties (e.g., stigma, social isolation, CSBD) could predispose individuals to seek partners online and then manifest in risky sexual behaviors. Engaging in online dating may in turn cause secondary adverse mental health outcomes such as depression or sexualized drug use. Identifying psychological and situational risk factors associated with use of DAs may facilitate a better understanding of mental health concerns among MSM. DAs may also have a positive impact on the social functioning of MSM in terms of greater availability of sexual or romantic partners, an increase in self-acceptance, and self-confidence. Despite some advantages, online dating seems to be associated with many severe threats in the area of mental health. Because of this, future studies should also focus on the development of prevention and therapeutic interventions relevant to the MSM group and their patterns of geosocial-networking app use.

## Author Contributions

KO and MG developed the idea for the paper and prepared the outline. KO and KS prepared the literature review. KO, KS, KL, and MG participated in manuscript writing. All authors contributed to the article and approved the submitted version.

## Conflict of Interest

The authors declare that the research was conducted in the absence of any commercial or financial relationships that could be construed as a potential conflict of interest.
